# The Increased Expression of an Engrailed to Sustain Shell Formation in Response to Ocean Acidification

**DOI:** 10.3389/fphys.2020.530435

**Published:** 2020-12-01

**Authors:** Yukun Zhang, Zhaoqun Liu, Yanan Zong, Yan Zheng, Yinan Li, Zirong Han, Lingling Wang, Linsheng Song

**Affiliations:** ^1^Liaoning Key Laboratory of Marine Animal Immunology, Dalian Ocean University, Dalian, China; ^2^Liaoning Key Laboratory of Aquatic Animal Disease Prevention and Control, Dalian Ocean University, Dalian, China; ^3^Dalian Key Laboratory of Aquatic Animal Disease Prevention and Control Dalian Ocean University, Dalian, China; ^4^Functional Laboratory of Marine Fisheries Science and Food Production Processes, Qingdao National Laboratory for Marine Science and Technology, Qingdao, China

**Keywords:** engrailed, *Crassostrea gigas*, shell formation, ocean acidification, stress response

## Abstract

Engrailed is a transcription factor required in numerous species for important developmental steps such as neurogenesis, segment formation, preblastoderm organization, and compartment formation. Recent study has proved that engrailed is also a key gene related to shell formation in marine bivalves. In the present study, the expression pattern of an engrailed gene (*Cg*engrailed-1) in Pacific oyster *Crassostrea gigas* under CO_2_-driven acidification was investigated to understand its possible role in the regulation of shell formation and adaptation to ocean acidification (OA). The open reading frame (ORF) of *Cg*engrailed-1 was obtained, which was of 690 bp encoding a polypeptide of 229 amino acids with a HOX domain. Phylogenetic analysis indicated that the deduced amino acid sequence of *Cg*engrailed-1 shared high homology with other engraileds from *Drosophila melanogaster*, *Mizuhopecten yessoensi*, and *Crassostrea virginica*. The mRNA transcripts of *Cg*engrailed-1 were constitutively expressed in various tissues with the highest expression levels detected in labial palp and mantle, which were 86.83-fold (*p* < 0.05) and 75.87-fold (*p* < 0.05) higher than that in hepatopancreas. The mRNA expression of *Cg*engrailed-1 in mantle decreased dramatically after moderate (pH 7.8) and severe (pH 7.4) acidification treatment (0.75- and 0.15-fold of that in control group, *p* < 0.05). The results of immunofluorescence assay demonstrated that the expression level of *Cg*engrailed-1 in the middle fold of mantle increased significantly upon moderate and severe acidification treatment. Moreover, after the oyster larvae received acidification treatment at trochophore stage, the mRNA expression levels of *Cg*engrailed-1 increased significantly in D-shape larvae stages, which was 3.11- (pH 7.8) and 4.39-fold (pH 7.4) of that in control group (*p* < 0.05). The whole-mount immunofluorescence assay showed that *Cg*engrailed-1 was mainly expressed on the margin of shell gland, and the periostracum in trochophore, early D-shape larvae and D-shape larvae in both control and acidification treatment groups, and the intensity of positive signals in early D-shape larvae and D-shape larvae increased dramatically under acidification treatment. These results collectively suggested that the expression of *Cg*engrailed-1 could be triggered by CO_2_-driven acidification treatment, which might contribute to induce the initial shell formation in oyster larvae and the formation of periostracum in adult oyster to adapt to the acidifying marine environment.

## Introduction

Engrailed is a homeodomain transcription factor which is involved in plentiful species for prime developmental period, and its evolution is known to be closely related to the evolution of metazoan body plan (Loomis et al., 1996; Iwaki and Lengyel, 2002; Byrne et al., 2005). The first engrailed was characterized from *Drosophila melanogaster* (Eker, 1929). Since then, lots of homologues of engrailed have been identified from various animal groups including mammals, annelids, arthropods, insects, echinoderms, and chordates (Fjose et al., 1985; Joyner et al., 1985; Holland et al., 1997; Lowe and Wray, 1997; Ikuta, 2017). There are two members included in the engrailed family of vertebrates, engrailed-1 and engrailed-2 (Joyner and Martin, 1985; Matsuura et al., 2010). The sequence of engrailed contains a conserved HOX domain, which is regarded as an important DNA-binding site for transcriptional regulation in crucial developmental processes (Desplan et al., 1985; Patel et al., 1989). The HOX domain of engrailed can bind to DNA mainly relying on the helix-turn-helix structure (two α-helixes connected by a β-corner), in which one of the α-helix combines some hydrogen bonds and hydrophobic interactions to special chains and groups in the major groove of DNA (Piper et al., 1999). Engrailed is also able to bind directly to the eukaryotic translation initiation factor 4E, via a sequence located in the *N*-terminus (Nedelec et al., 2004).

Engrailed in invertebrates share similar sequence features with their homologues in mammals, which also contain a conserved HOX domain. Engraileds identified from invertebrates are mainly responsible for body segmentation (Tucker-Kellogg et al., 1997). For example, engrailed from arthropods controlled the liner boundary between the anterior and posterior compartments in the wings (Morata and Lawrence, 1975; Poole et al., 1985). Recently, several engraileds have also been characterized from molluscs, which are found to be involved in shell formation (Miyazaki et al., 2010). It was reported that engrailed participated in shell formation in larval and adult molluscs through separate ways. As for the larval of molluscs, engrailed might take part in shell biosynthesis process by establishing compartment boundaries between embryonic domains (Nederbragt et al., 2002). Molluscan engraileds were mainly expressed in shell formation cells around the shell anlagen (Moshel et al., 1998; Kin et al., 2009), and at the edge of the embryonic shell, all of which were implied as the boundary of initial shell in larvae (Lawrence and Struhl, 1996). Besides, engrailed and another regulatory gene dpp were proposed to control the development of molluscan shell by confining the boundary of the larval shell, whose expression level remained high until umbo stage (Kin et al., 2009; Huan et al., 2013). Unlike larval stage, engrailed took part in shell formation in adult molluscs by regulating matrix protein expressions in mantles (Takeuchi and Endo, 2006; Zhou et al., 2010). It was reported that engraileds in adult nautilus were expressed in the marginal cells of the shell field in mantle (Baratte et al., 2007). In snail *Ilyanassa obsoleta*, the engrailed was detected in the shell gland progenitor and progeny, and the expression of engrailed mRNA was also observed in the shell plate (Moshel et al., 1998). These findings evidence that engrailed is crucial for shell formation in both larval and adult molluscs, and the underlying molecular mechanism deserves a better exploration.

Oysters are benthic species native to the coastal area worldwide. In recent years, the shell formation process in oyster is severely inhibited owing to the OA caused by excessive emission of greenhouse gas such as CO_2_ and CH_4_ (Waldbusser and Salisbury, 2014; Wang et al., 2017b). In the present study, the cDNA sequence of an previously reported engrailed, named as *Cg*engrailed-1, was cloned from the Pacific oyster *Crassostrea gigas* to (1) understand the molecular feature of *Cg*engrailed-1, (2) investigate the expression level of *Cg*engrailed-1 during larval developmental stages and in various tissues of adult oysters, and (3) explore the alteration of *Cg*engrailed-1 expression in the oysters under CO_2_-driven acidification and its possible role in the regulation of shell formation and adaptation to ocean acidification (OA).

## Materials and Methods

### Oyster Breading, Acidification Treatment and Sample Collection

The Pacific oysters *C. gigas* (about 2-year old, averaging 150 mm in shell length) were collected from a local farm in Dalian, Liaoning Province, China, and maintained in the aerated seawater at 20°C for 7 days before processing. The eggs and sperms were scraped from different adult oysters and mixed together for fertilization. After fertilization, the developing embryos were continuously observed by microscope until the second polar body was formed about 90 min post fertilization. Then, the embryos were transferred into an aquaculture tank containing 100 L aerated seawater of 20°C (Zhang et al., 2019).

Before acidification treatment on oyster larvae, trochophore were collected at 12 h post fertilization (hpf) according to previous description (Kin et al., 2009), and randomly divided into three groups. The remaining larvae were then transferred into three tanks containing seawater at a pH level of 8.10 ± 0.05 (control group), 7.80 ± 0.05 (moderate acidification treatment group) and 7.40 ± 0.05 (severe acidification treatment group), respectively. After acidification treatment, the larvae were collected at 15 hpf (early D-shape larvae) and 19 hpf (D-shape larvae), respectively. Samples for RNA extraction were added with 1 mL of Trizol reagent (Invitrogen, United States) in each tube. Larvae for whole-mount immunofluorescence assay were first relaxed by gradual addition of 7.5% MgCl_2_, followed by fixation in fresh 4% paraformaldehyde (PFA) in 0.01 M phosphate buffer saline (PBS) (0.2 g KCl, 8.0 g NaCl, 2.9 g Na_2_HPO_4_⋅12H_2_O, 0.2 g KH_2_PO_4_, pH 7.40) at 4°C for 3 h and washed three times (15 min for each time) with pre-cold PBS. After fixation, the samples were dehydrated and stored in pure methanol at −20°C for the subsequent whole-mount immunofluorescence assay.

As for the acidification treatment on adult oysters, 27 oysters were equally divided into three groups. Oyster without any treatment were designated as the control group (pH 8.10 ± 0.05), while the other bathed in acidified seawater at a pH level of 7.80 ± 0.05 and 7.40 ± 0.05 were employed as the moderate acidification treatment group and severe acidification treatment group (Wang et al., 2016). The mantles of three groups were collected at 7th day post treatment and every mantle sample collected from three oysters as one replicate. In addition, there are three replicates were conducted for each assay group. Samples for RNA extraction were added with 1 mL of Trizol reagent in each tube (Invitrogen, United States) and frozen immediately in liquid nitrogen. Tissues for sectioning and immunofluorescence assay were firstly fixed with Bouin’s solution (formalin 10%, picric acid saturated solution in distilled water, and glacial acetic acid, 15:5:1) for 24 h and then dehydrated with gradient ethanol.

The pH value of the acidification treatment group was controlled using an acidometer (AiKB, Qingdao, China). Total alkalinity was determined by end-point titration of 25 mmol L^–1^ HCl on 50 mL samples. The pH value, total alkalinity and partial press of CO_2_ (pCO_2_) were measured in the experiment. The total alkalinity in the pH 8.1, pH 7.8 and pH 7.4 groups was 2849.1 ± 14.5, 2416.3 ± 125.1, and 2110.6 ± 75.3 μeq kg^–1^, respectively. And the partial pressure of CO_2_ was about 658.1 ± 11.0, 1217.3 ± 11.6, and 2268.4 ± 50.1 ppm in pH 8.1, pH 7.8 and pH 7.4 treatment groups, respectively (Zhang et al., 2019). All the animal-involving experiments of this study were approved by the Ethics Committee of Dalian Ocean University.

### RNA Extraction and cDNA Synthesis

Total RNA was isolated from oyster tissues using Trizol reagent according to the standard protocol (Invitrogen). RNA concentration was measured on a NanoDrop reader (Saveen & Werner ApS, Denmark), and the integrity and purity of RNA was examined by running in 1.0% agarose gel. The total RNA was then treated with DNaseI (promega) to remove trace DNA contamination. The synthesis of the first-strand cDNA was carried out with Promega M-MLV RT with oligo (dT)-adaptor priming according to the manufactory’s protocol. The synthesis reaction was performed at 42°C for 1 h, terminated by heating at 95°C for 5 min. The cDNA mix was diluted to 1:40 and stored at −80°C for subsequent SYBR Green Fluorescent Quantificational real-time PCR.

### Gene Cloning and Sequence Analysis

An engrailed (CGI_10012208) was identified from oyster which was designated as *Cg*engrailed-1 (Huang et al., 2015). The open reading frame (ORF) of *Cg*engrailed-1 was cloned from cDNA library using specific primers (P1 and P2, Table 1). The PCR procedure of *Cg*engrailed-1 was as follows: 5 min at 95°C; 30 cycles at 94°C for 30 s, 56°C for 1 min, 72°C for 40 s, and 72°C for 10 min. The PCR product was gel-purified, cloned into PMD 19-T simple vector (Takara), and confirmed by DNA sequencing.

**TABLE 1:** Sequences of the primers used in this study.

Primer	Sequence (5′–3′)
Clone primers	
P1 (*Cg*engrailed-1F)	ATGAAGATTTGGTAATTAGATCGAG
P2 (*Cg*engrailed-1R	AACTGTTACTGTTGAATGGTTATAAAG
Expression primers	
P3 (*Cg*engrailed-1F_e_)	CGCGGATCCATGGATGTTAAACAAAATAACGCG
P4 (*Cg*engrailed-1R_e_)	CCCAAGCTTTGACTCGCCTTCATCCGA
RT-PCR primers	
P5(*Cg*EF-RT-F)	AGTCACCAAGGCTGCACAGAAAG
P6(*Cg*EF-RT-R)	TCCGACGTATTTCTTTGCGATGT
P7 (*Cg*engrailed-1RT-F)	GCCATTGGTTATCGGCATTTT
P8 (*Cg*engrailed-1RT-R)	AGAGTTGGGAGTCTGATGGTGAAA

The homology searches of the cDNA sequence and protein sequence of *Cg*engrailed-1 was conducted with BLAST algorithm at the National Center for Biotechnology Information^1^. The deduced amino acid sequence was analyzed with the Expert Protein Analysis System^2^. The protein domain was predicted with the simple modular architecture research tool^3^ (SMART) version 5.1. Multiple sequence alignment of *Cg*engrailed-1 with other engraileds was created by the ClustalW multiple alignment program^4^ and multiple sequence alignment show program^5^.

### Quantitative Real-Time PCR

The expression levels of *Cg*engrailed-1 in different development stages and different tissues were measured by an ABI PRISM 7500 Sequence Detection System with a total volume of 25.0 μL, containing 12.5 μL of SYBR Green Mix (Takara), 0.5 μL of each primer (10 μmol L^–1^) (P5 and P6, Table 1), 2.0 μL of cDNA, and 9.5 μL of DEPC-water. The oyster elongation factor (EF) was used as internal control (P7 and P8, Table 1). Dissociation curve analysis of amplification products was performed to confirm that only one PCR product was amplified and detected. The comparative average cycle threshold method was used to analyze the mRNA expression level of the immune-related genes according to previous research (Zhang et al., 2008). All data were given in terms of relative mRNA expression using the 2^–Δ^ ^Δ^ ^*Ct*^ method (Yu et al., 2007).

### Recombinant Expression and Purification of *Cg*engrailed-1

The *Cg*engrailed-1 was induced and expressed through the prokaryotic expression system. The ORF fragment of *Cg*engrailed-1 was gained from the PCR reaction with pairs of primers (P3 and P4, Table 1). The PCR products and the pET-30a expression vector were linearized with double enzyme digestion, and then the PCR products and linearized expression vector were recombined to a complete vector through T4 ligase system. The DNA sequence combined to pET-30a expression vector was confirmed by DNA sequencing. The valid recombinant plasmid was extracted and transformed into *Escherichia coli* transetta DE3. Positive transformants were cultured in LB medium at 37°C, 120 rpm incubator. When the OD_600_ value of bacteria solution reached 0.4–0.6, the bacteria were incubated for additional 8 h with the induction of Isopropyl β-D-thiogalactoside (IPTG) at the final concentration of 1 mM. The recombinant protein of *Cg*engrailed-1 labeled with 6× His-tag at the *C*-terminal was purified by Ni^+^ affinity chromatography, and desalted by extensive dialysis. The purified objective protein (r*Cg*engrailed-1) was concentrated and stored at −80°C (Zong et al., 2019).

### Preparation of Polyclonal Antibodies Against *Cg*engrailed-1

Six-week-old mice were immunized with r*Cg*engrailed-1 to acquire polyclonal antibodies following the description of previous study (Cheng et al., 2006). Briefly, 100 μL r*Cg*engrailed-1 (0.3 mg mL^–1^) was emulsified with 100 μL complete Freund’s adjuvant (Sigma, United States) to immunize each mouse by subcutaneous implantation. The second and third injections were performed on the 14th and 21th day with incomplete Freund’s adjuvant (Sigma, United States). The fourth injection was performed with 100 μL r*Cg*engrailed-1 on the 28th day. The anti-r*Cg*engrailed-1 serum was obtained on the 36th day and stored at −80°C before use.

### Western Blot Analysis of *Cg*engrailed-1

The specificity of polyclonal antibody against r*Cg*engrailed-1 and natural *Cg*engrailed-1 (from mantles) was verified by western blot assay. Briefly, r*Cg*engrailed-1 and natural *Cg*engrailed-1 was separated by 12% SDS-PAGE, and electrophoretically transferred onto a nitrocellulose membrane. The membrane was washed three times with TBS containing 0.1% Tween 20 (TBST), blocked by 5% skimmed milk (100 mL TBST, 5 g skimmed milk) at 4°C overnight, and then incubated with 1:500 diluted polyclonal antibodies against r*Cg*engrailed-1 at 37°C for 3 h. After three times of washing with TBST, the membrane was incubated with 1:3,000 diluted secondary antibody Goat-anti-mouse IgG conjugated with HRP (ABclonal) at 37°C for 2 h. After the final three times of washing with TBST, the protein bands were developed by using Super ECL Detection Reagent (Sigma-Aldrich) for one minute, capture image and take picture by Amersham Imager 600 system (GE Healthcare, United States).

### Whole-Mount Immunofluorescence Assay

The whole-mount immunofluorescence assay was conducted as previously described (Thisse and Thisse, 2008; Liu et al., 2015) with minor modification. In brief, the larvae stored in pure methanol were rehydrated by PBS and washed three times with PBST (PBS with 0.1% Tween20). The shells of D-shape larvae were decalcified in 25 mmol^–1^ EDTA in PBS for 30 min, rinsed in PBST for 3 × 15 min, and blocked overnight in the blocking buffer (10% normal goat serum, 0.25% fetal bovine serum albumin, 1% TritonX-100 and 0.03% sodium azide in PBS). Then, the larvae were incubated with the primary antibodies (1:500 dilutions in blocking buffer) at 4°C for 3–4 days. These specimens were then washed for 3 × 15 min in PBST and incubated with goat anti-rat IgG conjugated to Alexa Fluor 488 (1:2,000 dilutions in blocking buffer; Invitrogen, Carlsbad, CA, United States) for 1 day at room temperature. After incubation in secondary antibodies, all the specimens were washed in PBST for 3 × 20 min and then mounted in 50% glycerol in PBS. The specimens incubated with pre-immune serum added with secondary antibodies were selected as negative control (Liu et al., 2015). All specimens were examined as whole-mount using the Zeiss Laser-Scanning Confocal Microscopy System LSM 710 (Zeiss, Jena, Germany).

### Mantle Paraffin Embedding, Sectioning and Immunofluorescence Histochemical Assay

The oyster mantles from control group and acidification treatment group were fixed in Bouin’s solution for 24 h and decolorized with 75% (v/v) ethanol. Then, the fixed samples were embedded in paraffin, and sectioned at 6 μm thickness. The slides were treated to remove the paraffin and rehydrated. Soak the slides in 0.01 M citrate buffer solution, conduct antigen renaturation in autoclave at 120° C for 6 min, and then cool it to room temperature naturally. After cooling down at room temperature, the slides were prepared for immunohistochemistry staining. The slides were first blocked in BSA, and *Cg*engrailed-1 polyclonal antibody was used as primary antibody (diluted at 1:500 in blocking buffer). Alexa Fluor 488 labeled goat-anti-mouse (diluted at 1:1,000, Abclonal, College Park, MD, United States) was used as secondary antibody. Finally, the slides were incubated with 4′, 6-diamidine-2-phenylindole dihydrochloride (DAPI, 10 mg mL^–1^, diluted in PBS by 1:2,000) (Roche, Nutley, NJ, United States) for 5 min. After three times of washing, 50% glycerol was added on the slides before observation under Axio Imager A2 microscope (Carl Zeiss, Germany).

### Statistical Analysis

All the data were shown as mean ± S.D. The two-samples Student’s tested were performed for the comparisons between groups. Multiple group comparisons were executed by one-way ANOVA and followed by a Turkey multiple group comparison tests using Statistical Package for Social Sciences (SPSS) 16.0 Software. The difference was considered as significant at *p* < 0.05.

## Results

### The Sequence Features and Phylogenetic Relationship of *Cg*engrailed-1

**FIGURE 1:**
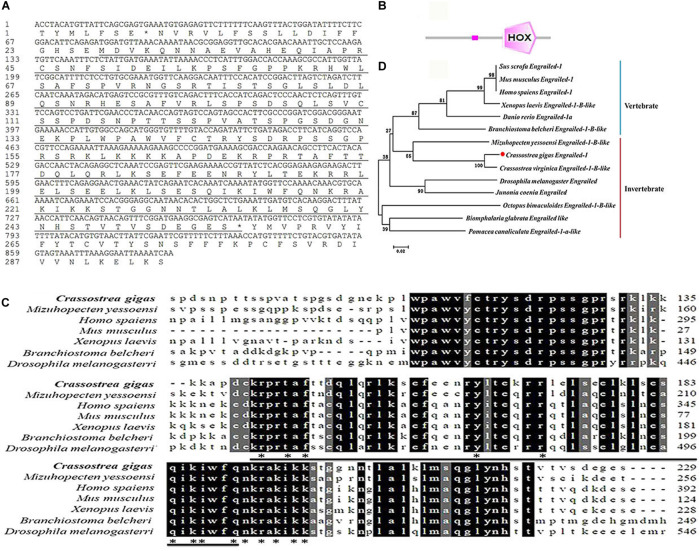
Bioinformatics analysis of *Cg*engrailed-1. **(A)** The cDNA sequence and the putative protein of *Cg*engrailed-1 which is marked by black underline. **(B)** The typical HOX domain predicted by SMAT analysis. **(C)** Multiple sequence alignment of the *Cg*engrailed-1. Multiple sequence alignment of *Cg*engrailed-1 with known engraileds from various species including *C. gigas* (XP_011414452.1), *M. yessoensi* (XP_021372813.1), *Homo sapiens* (NP_001417.3), *Mus musculus* (NP_034263.2), *Xenopus laevis* (XP_018094123.1), *Branchiostoma belcheri* (XP_019636788.1), and *Drosophila melanogaster* (NP_523700.2). **(D)** Phylogenetic analysis of *Cg*engrailed-1. Fourteen engrailed sequences from different species were obtained from GenBank on NCBI, including *Sus scrofa* (XP_003133330.2), *Mus musculus* (NP_034263.2), *Homo sapiens* (NP_001417.3), *Xenopus laevis* (XP_018094123.1), *Danio rerio* (NP_571120.2), *Branchiostoma belcheri* (XP_019636788.1), *M. yessoensi* (XP_021372813.1), *C. gigas* (XP_011414452.1), *C. virginica* (XP_022294249.1), *Drosophila melanogaster* (NP_523700.2), *Junonia coenia* (AAD08923.1), *Octopus bimaculoides* (XP_014774611.1), *Biomphalaria glabrata* (XP_013080601.1), and *Pomacea canaliculata* (XP_025083924.1). ClustalX software was used to perform the multiple sequence alignment of the fourteen engrailed sequences. The neighbor-joining (NJ) tree of *Cg*engrailed-1 was constructed with Mega 6.0 software.

The ORF of a previously reported engrailed gene (*Cg*engrailed-1, CGI_10012208) was identified from the Pacific oyster *C. gigas*, which was of 690 bp encoding a polypeptide of 229 amino acids (Figure 1A). The putative molecular weight of *Cg*engrailed-1 was 25.70 kDa and its theoretical isoelectric point (pI) was 9.62. SMART analysis showed that there was a HOX domain (from Glu141 to Asn203) and a low expression region in *Cg*engrailed-1 (Figure 1B).

Results of multiple sequences alignment revealed that the deduced amino acid sequence of *Cg*engrailed-1 shared 48.50% identity with that from *Mizuhopecten yessoensi*, 41.30% identity with that from *Xenopus laevis*, 40.50% identity with that from *Branchiostoma belcheri*, 37.90% identity with that from *Mus musculus*, 27.50% identity with that from *Homo sapiens*, and 19.60% identity with that from *Drosophila melanogaster* (Figure 1C). A total of 14 engraileds from various vertebrate and invertebrate species were employed in phylogenetic analysis. There were two major distinct branches in the phylogenetic tree, the vertebrate and invertebrate branches. *Cg*engrailed-1 was firstly gathered with that from *C. virginica* and then clustered with those from *M. yessoensis* and other molluscs, and finally clustered with those from vertebrates (Figure 1D).

### Preparation of Recombinant Protein and Polyclonal Antibodies of *Cg*engrailed-1

**FIGURE 2:**
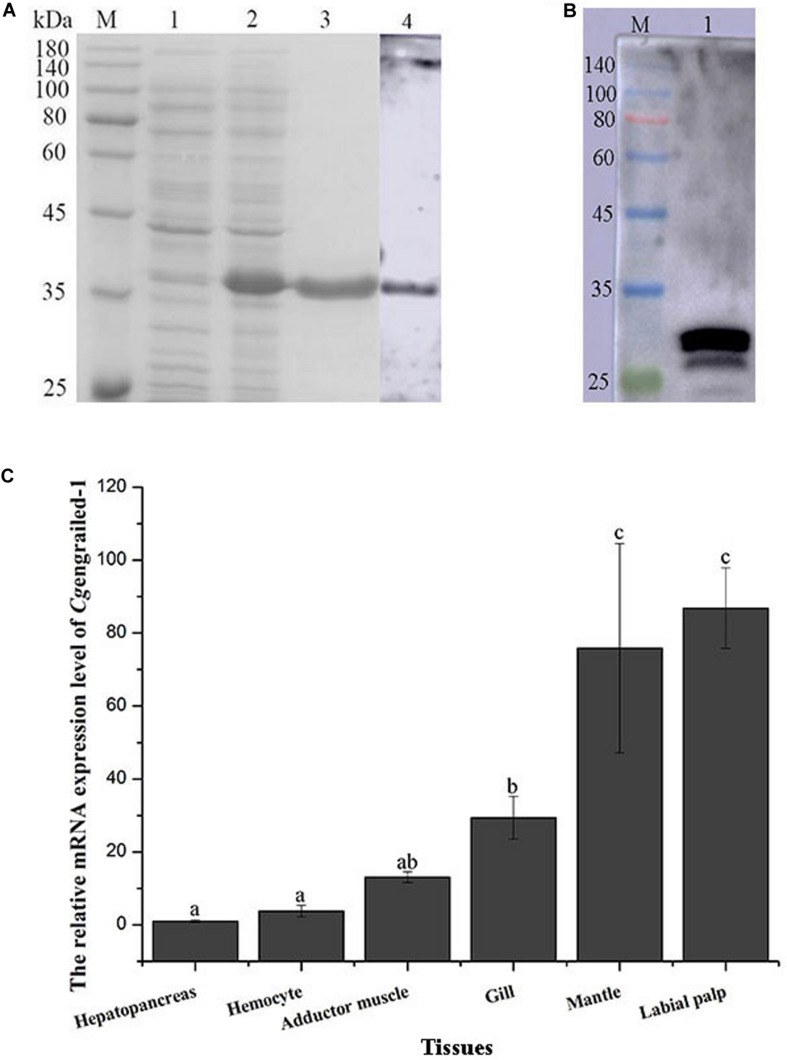
**(A)** SDS-PAGE and western blot analysis of r*Cg*engrailed-1. Lane M: protein molecular standard; Lane 1: Proteins form *E. coli* without IPTG induction; Lane 2: positive transformant induced by IPTG; Lane 3: purified r*Cg*engrailed-1; Lane 4: western blot analysis of the polyclonal antibody against r*Cg*engrailed-1. **(B)** Western blot analysis of *Cg*engrailed-1 from mantle. Lane M: protein molecular standard; Lane 1: natural protein isolated from mantle. **(C)** *Cg*engrailed-1 mRNA expression level in different tissues. Each value was shown as mean ± SD (*N* = 3), and statistical significance (*p* < 0.05) was marked by different letters (a–c).

The ORF fragment of *Cg*engrailed-1 was inserted into the pET-30a vector to build the recombinant plasmid and transferred into the expression bacterial strain (*E. coli* Transetta, DE3). After IPTG induction, the purified recombinant protein of r*Cg*engrailed-1 was ascertained by the 12% SDS-PAGE through electrophoresis (Figure 2A). There is one single band about 35 kDa detected by SDS-PAGE, which was in consistent with the predicted molecular weight of r*Cg*engrailed-1 (Figure 2A, lane 3). There was no corresponding band in the cell lysate without induction (Figure 2A).

The purified r*Cg*engrailed-1 was used to immune the female mice to prepare the polyclonal antibody. The polyclonal antibody specificity of the r*Cg*engrailed-1 was verified by western blot. There was only a distinct band detected, whose molecular weight was in accordance with that of r*Cg*engrailed-1 (Figure 2B). Meanwhile, no distinct band was observed in the negative control.

### The Distribution of *Cg*engrailed-1 mRNA Transcripts in Oyster Tissues

The expression level of *Cg*engrailed-1 mRNA in oyster tissues was detected by quantitative real-time PCR. The mRNA transcripts of *Cg*engrailed-1 were widely expressed in all the tested tissues including hepatopancreas, hemocytes, adductor muscle, gill, mantle and labial palp (Figure 2C). The highest expression level of *Cg*engrailed-1 transcripts was observed in labial palp, which was approximately 86.83-fold of that in hepatopancreas (*p* < 0.05). In addition, the expression level of *Cg*engrailed-1 transcripts in mantle and gill was 75.87-fold (*p* < 0.05) and 28.52-fold (*p* < 0.05) higher than that in hepatopancreas, respectively.

### The Expression of *Cg*engrailed-1 mRNA in Oyster Larvae Upon Acidification Treatment

**FIGURE 3:**
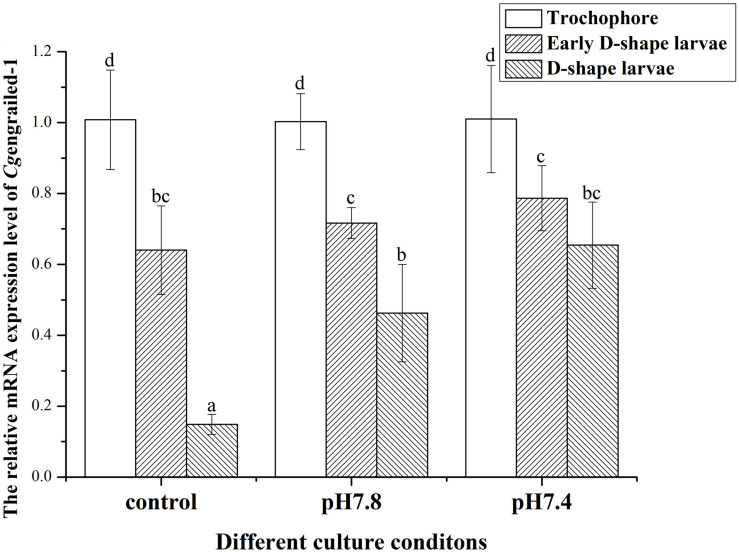
Changes of *Cg*engrailed-1 mRNA expression in trochophore, early D-shape larvae and D-shape larvae under acidification treatment. Each value was shown as mean ± SD (*N* = 3), and statistical significance (*p* < 0.05) was marked by different letters (a–c).

In the control (pH 8.1) group, the mRNA expression levels of *Cg*engrailed-1 decreased dramatically at early D-shape larvae and D-shape larvae stages, which were 0.64- and 0.15-fold compared to trochophore (*p* < 0.05). After the larvae received acidification treatment at trochophore stage, the mRNA expression levels of *Cg*engrailed-1 increased significantly in D-shape larvae stage, which was 3.11- (pH 7.8, moderate acidification treatment) and 4.39-fold (pH 7.4, severe acidification treatment) of that in control group (*p* < 0.05). No significant change of *Cg*engrailed-1 mRNA was observed in early D-shape larvae after acidification treatment, which was 1.12- (pH 7.8) and 1.23-fold (pH 7.4) of that in control group (*p* < 0.05, Figure 3).

**FIGURE 4:**
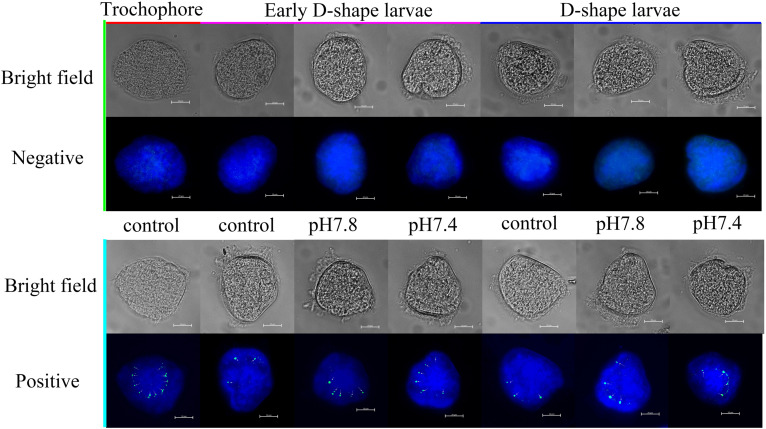
Whole-mount immunofluorescence assay of *Cg*engrailed-1 expression in oyster larvae. The expression of *Cg*engrailed-1 in oyster larvae at trochophore, early D-shape larvae and D-shape larvae stages upon moderate (pH 7.8) and severe (pH 7.4) acidification treatments were determined by whole-mount immunofluorescence assay. Mouse negative serum was employed in the negative control group. Nucleus was stained with DAPI and shown in blue; Green point indicated the positive signal which was depicted by little white arrows.

The expression of *Cg*engrailed-1 in oyster larvae in response to the acidification treatment was also measured by whole-mount immunofluorescence assay. In the control group (pH 8.1), the positive signals (the green point) were observed on the margin of shell gland or periostracum in trochophore, early D-shape larvae and D-shape larvae, and positive signals gradually decreased following the developmental progresses. After moderate or severe acidification treatment, the positive signals were observed on the same location of oyster larvae. No signal was detected in the negative group, indicating the specificity of polyclonal antibody and the reliability of the present results. In trochophore, lots of small and dense positive signals were observed on the dorsal edge of the periostracum. Once the oyster larvae received acidification treatment from trochophore stage, the positive signals in both early D-shape larvae and D-shape larvae were stronger than that in the control group (Figure 4).

### The Expression Level of *Cg*engrailed-1 mRNA in Oyster Mantle Upon Acidification Treatment

**FIGURE 5:**
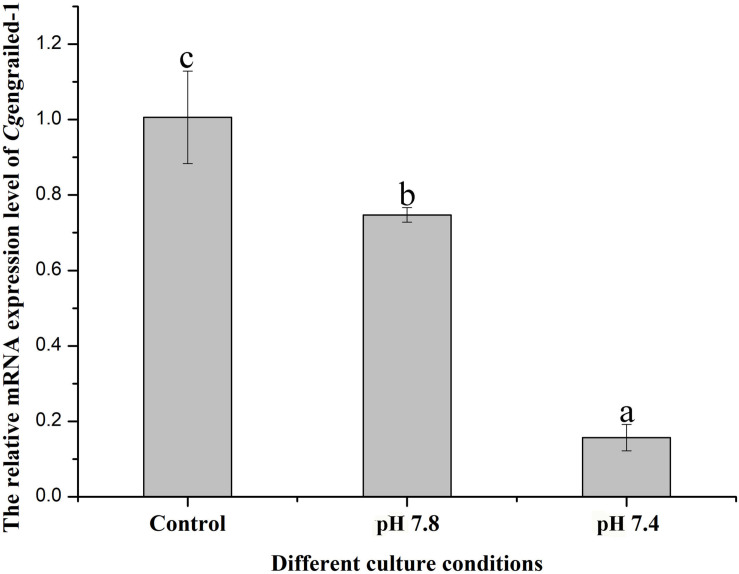
Changes of *Cg*engrailed-1 expression in adult oyster mantle upon acidification treatment. The mRNA expression of *Cg*engrailed-1 in mantle tissue of adult oyster upon moderate (pH 7.8) and severe (pH 7.4) acidification treatments were determined by quantitative real-time PCR. Each value was shown as mean ± SD (*N* = 3), and statistical significance (*p* < 0.05) was marked by different letters (a–c).

The expression level of *Cg*engrailed-1 mRNA in the mantle of adult oysters after different acidification treatment was determined by quantitative real-time PCR. After the adult oysters received moderate (pH 7.8) and severe acidification (pH 7.4) treatment for one week, the mRNA expression level of *Cg*engrailed-1 in mantle decreased significantly, which were 0.75- and 0.15-fold (*p* < 0.05) of that in the control (pH 8.1) group. The decreasing trend in pH 7.4 group was more obvious than that in pH 7.8 group (Figure 5).

**FIGURE 6:**
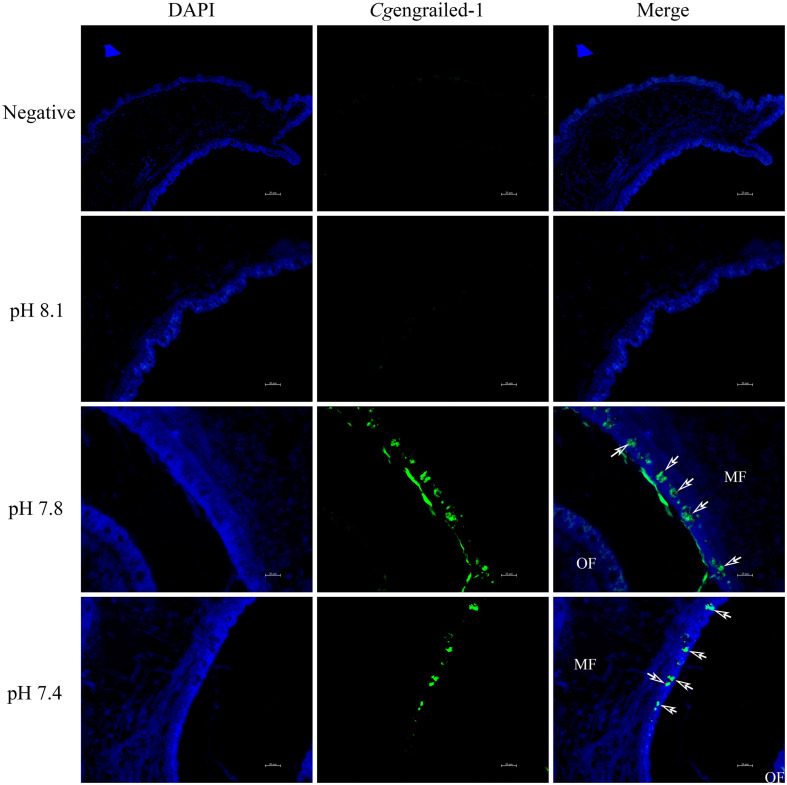
Detection of *Cg*engrailed-1 in oyster mantle of upon acidification treatment by immunofluorescence assay. Nucleus was stained with DAPI and shown in blue; anti-*Cg*engrailed-1 coupled to Alexa fluor-488 was shown in green. The white arrow indicated the positive signal. MF, middle fold of mantle edge; OF, outer fold of mantle edge.

The expression of *Cg*engrailed-1 in mantle responsive to experimental acidification treatment was further examined by immunofluorescence assay. No positive signal was observed in the negative and control (pH 8.1) groups. When adult oysters received acidification treatment, positive signals were detected in the middle fold of mantle, and the signal intensity in moderate acidification treatment group was significantly stronger than that in severe acidification treatment group (Figure 6).

## Discussion

Marine bivalves such as oysters and mussels secret matrix proteins and deposit calcium carbonate to form the calcified shells as a supporting skeleton to protect their soft bodies (Zhang et al., 2012; Hendriks et al., 2015), which is thought to be one of the key factors that trigger the expansion of bivalves at the dawn of the Cambrian times (Marie et al., 2012). However, they are now in great danger due to the OA caused by excessive burning of fossil fuels (Orr et al., 2005; Waldbusser et al., 2015; Wang et al., 2017a), and there is urgent need for a better understanding of the shell formation mechanism in bivalves and their stress responses and adaptation modes to CO_2_-driven acidification. Previous study proved that engrailed, a transcription factor related to the evolution of the metazoan body plan, was also responsible for shell formation in bivalves. However, it has never been explored whether OA affects such process by interrupting the expression of engrailed. In the present study, the expression patterns of a previously identified engrailed upon experimental acidification treatment in both larval and adult oysters were explored, hoping to illustrate the involvement of engrailed in oyster shell formation and acidification adaptation.

In this study, the ORF of *Cg*engrailed-1 was cloned from *C. gigas*, which was of 690 bp encoding a polypeptide of 229 amino acids. *Cg*engrailed-1 contained a typical HOX domain (from Glu141 to Asn203) and a low expression region without signal peptide and transmembrane domain. In vertebrates, engrailed is a member of the homeodomain transcription factors which possess a HOX domain and take part in many developmental processes through combining DNA (Brunet et al., 2005). There were several predicted DNA binding residues identified in *Cg*engrailed-1, including Arg (143), Thr (146), Phe (148), Tyr (165), Arg (171), Gln (184), Lys (186), Ile (187), Gln (190), Asn (191), Arg (193), Lys (195), Lys (197), and Lys (198). These residues regulate transcription via combining some special groups on the DNA sequence to affect segmentation, neurogenesis and metamorphosis (Morgan, 2006). According to previous study, engrailed may play vital roles in embryonic development and limb development via regulating the active state of key factor Gli in the hedgehog signal pathway (Riddle et al., 1993; Simonnet et al., 2004). The inferred amino acid sequences of *Cg*engrailed-1 shared higher sequence identity with its homologues from other invertebrates than in vertebrates. Phylogenetic analysis showed that *Cg*engrailed-1 shared high homology with engraileds from *M. yessoensi* and *C. virginica*. These results indicated that *Cg*engrailed-1 belonged to the engrailed family, which exhibited the function of a transcription factor.

Previous studies have reported that engraileds are responsible for the set-up of compartment boundaries between embryonic domains in molluscan species such as *P. vulgate* and *C. gigas* (Nederbragt et al., 2002; Huang et al., 2015). It was found that molluscan engraileds were mainly expressed in the shell forming cells (Moshel et al., 1998) around the shell anlagen (Kin et al., 2009), and at the edge of the embryonic shell, all of which were implied as the boundary of initial shell in larvae (Lawrence and Struhl, 1996). In the present study, results of the whole-mount immunofluorescence assay showed that *Cg*engrailed-1 was mainly expressed at the margin of shell gland or periostracum of oyster larvae, which was the exact region that the shell boundary formed (Huang et al., 2015). These results demonstrated that *Cg*engrailed-1 might be involved in the early shell formation progress in oyster larvae. In addition, after the trochophore of oyster received moderate and severe acidification treatment, the mRNA and protein expressions of *Cg*engrailed-1 altered significantly. Both moderate and severe acidification treatment could prompt the expression of *Cg*engrailed-1 mRNA, and the increase was greater in the severe acidification treatment group (pH 7.4). Results of whole-mount immunofluorescence assay revealed that the positive signals (indicating *Cg*engrailed-1 expression) were observed at the margin of periostracum in trochophore, early D-shape larvae and D-shape larvae under acidification treatment. These results suggested that *Cg*engrailed-1 was involved in larval shell formation in oyster, and both moderate and severe acidification treatment could significantly prompt the expression of *Cg*engrailed-1. In order to relieve the negative effects imposed by acidification treatment, oyster larvae tended to induce the expression of *Cg*engrailed-1 to sustain the formation of shell boundary.

In addition, it was found that engrailed participated in shell formation in adult molluscs through different ways from that in larval molluscs. The mRNA transcripts of *Cg*engrailed-1 were highly expressed in mantle comparing with other tissues. Severe acidification treatment could significantly trigger the expression of *Cg*engrailed-1 in mantle. Moreover, some dispersive signals (indicating *Cgengrailed-1* expression) were detected at the epithelial cell of the mantle middle fold in moderate acidification treatment group, and the signals in pH 7.8 group were more intensive than those in pH 7.4 and control groups. The edge of oyster mantle consists of three folds, including inner fold, middle fold and outer fold (Supplementary Figure S1), and the middle fold was responsible for the formation of periostracum which was the major component of the molluscan shell (Saleuddin, 1979; Song et al., 2019). Previous study had constructed the biological model of periostracum secretion in bivalves, which indicated that the periostracum layer was initiated as a pellicle by basal cells located deep in the periostracal groove (Checa, 2000), and the outer epithelial cells of the middle fold in the mantle were responsible to lubricate and reinforce the pellicle in place (Waite, 1983). Some later research discovered that the outer epithelial cells of the middle fold also contained some membrane-bound vesicles (Richardson et al., 1981). In the present study, *Cg*engrailed-1 expression in the region close to periostracal groove was significantly increased upon acidification treatment, which suggested that *Cg*engrailed-1 was involved in the regulation of shell formation in adult oyster in response to acidification threat by modulating the synthesis of periostracum. These results indicated that *Cg*engrailed-1 might involve in shell formation in adult oyster by regulating the biosynthesis of periostracum in the epithelial cell in the middle fold of mantle. Acidification treatment could prompt the expression of *Cg*engrailed-1 in the middle fold of mantle to sustain the biosynthesis of periostracum in response to acidification stress.

In summary, the ORF of *Cg*engrailed-1 was cloned from oyster *C. gigas*, which contained a HOX domain and belonged to the homeodomain transcription factor family. *Cg*engrailed-1 was involved in the shell formation progress and the response of larval and adult oyster to the acidification stress through separate ways. The *Cg*engrailed-1 tended to increase expression to sustain the formation of shell boundary in oyster larvae, while it was prompted for the significant expression in the epithelial cell in the middle fold of mantle to trigger the biosynthesis of periostracum in adult oysters. Both larval and adult oysters were able to relieve the negative effects imposed by acidification treatment through increasing the expression of *Cg*engrailed-1.

## Data Availability Statement

The raw data supporting the conclusions of this article will be made available by the authors, without undue reservation.

## Ethics Statement

The animal study was reviewed and approved by the Ethics Committee of Dalian Ocean University.

## Author Contributions

YKZ, ZL, and LW conceived and designed the experiments. YKZ, YNZ, YL, ZH, and YZ performed the experiments. YKZ and ZL analyzed the data. LW and LS contributed reagents, materials, and analysis tools. YKZ, ZL, LW, and LS wrote the manuscript. All the authors read and approved the final manuscript.

## Conflict of Interest

The authors declare that the research was conducted in the absence of any commercial or financial relationships that could be construed as a potential conflict of interest.

## Footnotes

1 http://www.ncbi.nlm.gov/blast

2 http://www.expasy.org

3 smart.embl-heidelberg.de

4 http://www.ebi.ac.uk/Tools/clustalw2/

5 http://www.biosoft.net/sms/index.html

## Supplementary Material

Supplementary Figure 1 The structure of the edge of oyster mantle. The outer fold was close to the edge of the shell and the inner fold was close to the gill tissue. The black arrow indicated the expression of *Cg*engrailed-1 protein. MF, middle fold of mantle edge; OF, outer fold of mantle edge; PG, periostracal groove.
