# Supplementary figures and images for: The Increased Expression of an Engrailed to Sustain Shell Formation in Response to Ocean Acidification
Source: Front Physiol. 2020 Dec 1;11:530435. doi: 10.3389/fphys.2020.530435 (PMC7793958; doi:10.3389/fphys.2020.530435)

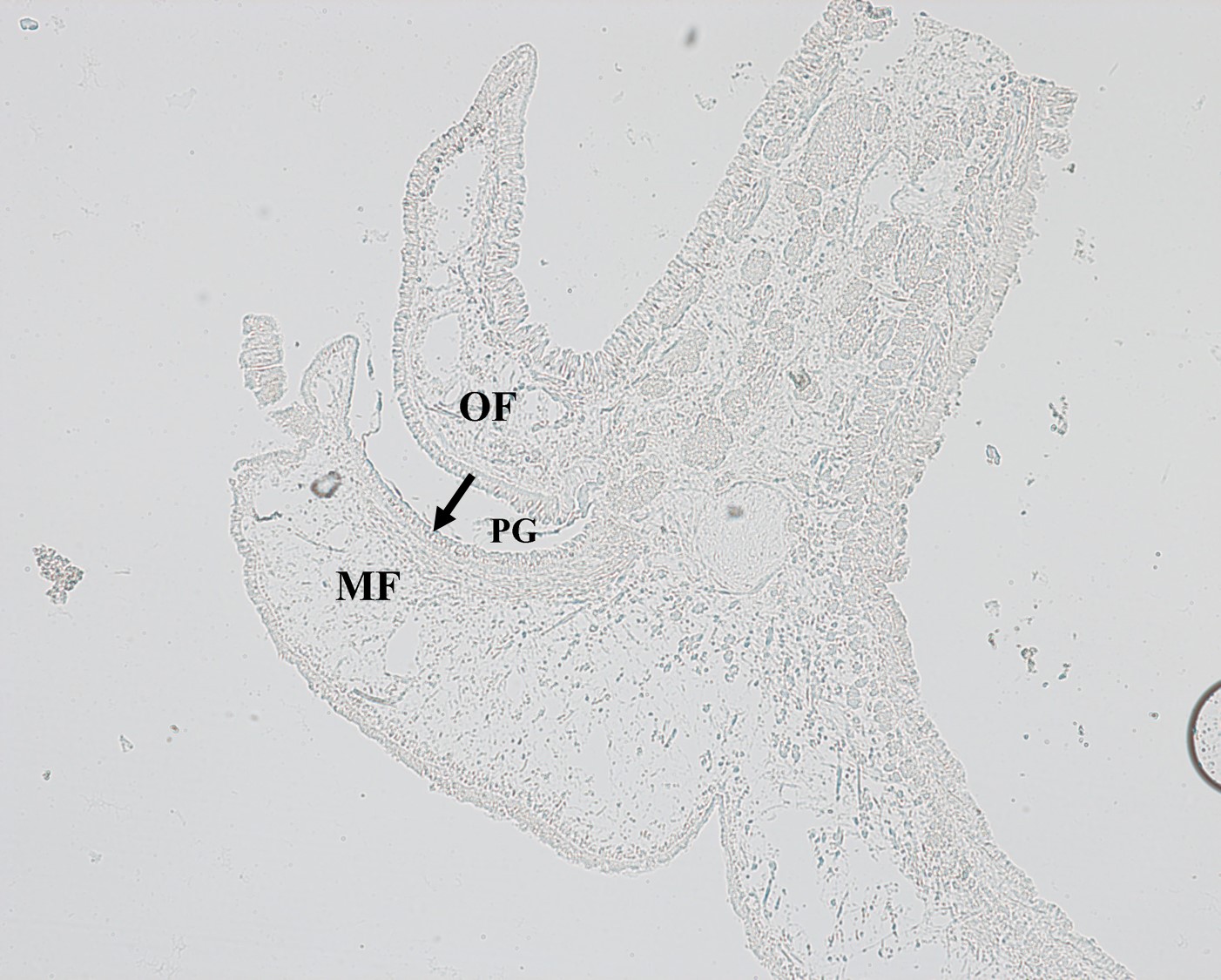

Supplement: Supplementary Figure 1 — The structure of the edge of oyster mantle. The outer fold was close to the edge of the shell and the inner fold was close to the gill tissue. The black arrow indicated the expression of Cgengrailed-1 protein. MF, middle fold of mantle edge; OF, outer fold of mantle edge; PG, periostracal groove. [file Image_1.JPEG]
