# Supplementary material for: The Increased Expression of an Engrailed to Sustain Shell Formation in Response to Ocean Acidification
Source: Front Physiol. 2020 Dec 1;11:530435. doi: 10.3389/fphys.2020.530435 (PMC7793958; doi:10.3389/fphys.2020.530435)
Supplement: Supplementary file 2 [file Table_1.DOCX]

**Table 1. Sequences of the primers used in this study**

| **Primer** | **Sequence (5'-3')** | |
| --- | --- | --- |
| **Clone primers** | |  |
| P1 (*Cg*engrailed-1F)  P2 (*Cg*engrailed-1R | | ATGAAGATTTGGTAATTAGATCGAG  AACTGTTACTGTTGAATGGTTATAAAG |
| **Expression primers**  P3 (*Cg*engrailed-1F_e_)  P4 (*Cg*engrailed-1R_e_)  **RT-PCR primers** | | CGCGGATCCATGGATGTTAAACAAAATAACGCG  CCCAAGCTTTGACTCGCCTTCATCCGA |
| P5(*Cg*EF-RT-F) | | AGTCACCAAGGCTGCACAGAAAG |
| P6(*Cg*EF-RT-R)  P7 (*Cg*engrailed-1RT-F)  P8 (*Cg*engrailed-1RT-R) | | TCCGACGTATTTCTTTGCGATGT  GCCATTGGTTATCGGCATTTT  AGAGTTGGGAGTCTGATGGTGAAA |
